# The development and testing of a single-arm feasibility and acceptability study of a whole foods diet intervention for adults with prediabetes and their offspring

**DOI:** 10.1186/s40814-024-01554-9

**Published:** 2024-10-23

**Authors:** Nadia M. Sneed, Raegan Kelley, Haley Turner, Mariann R. Piano, Chloe Dagostino, Ashley Sellers, Kemberlee Bonnet, David Schlundt, Laura E. Adams, William J. Heerman

**Affiliations:** 1 Center for Research and Scholarly Development, Vanderbilt University School of Nursing, 461 21st Avenue S, Nashville, TN 37240 USA; 2 Vanderbilt Student Health Center, 1210 Stevenson Center LN, Nashville, TN 37232 USA; 3 Department of Psychology, Vanderbilt University, Nashville, TN USA; 4 Department of Pediatrics, Vanderbilt University Medical Center, 2145 Belcourt Avenue, Nashville, TN 37212 USA

**Keywords:** Nutrition, Prediabetes, Type 2 diabetes risk, Dietary pattern, Dietary Guidelines for Americans, Family-based intervention, Feasibility study

## Abstract

**Background:**

Diet is considered a first-line treatment option for prediabetes, a condition that affects 96 million United States (U.S.) adults. Diet patterns that prioritize whole foods (e.g., Mediterranean) are currently recommended to treat prediabetes. However, no studies have tested whether a U.S.-style diet pattern that prioritizes whole foods can be used to treat prediabetes. The purpose of this study was to assess the feasibility and acceptability of a whole foods diet for adults with prediabetes and their offspring prior to conducting a larger clinical trial.

**Methods:**

A 2-week single-arm pre-post experimental controlled-feeding intervention of a 2020–2025 Dietary Guidelines for Americans adapted whole foods diet (e.g., primarily focused on foods that have undergone limited processing or refinement) was conducted in adults (25–59 years) with prediabetes and their biological offspring (6–17 years). Families received 2 weeks of menus and grocery delivery and weekly counseling by a registered dietitian. Families were invited to attend an optional focus group session. Feasibility was based on having a ≥ 50% family completion rate with ≥ 80% completion of study outcomes. Measures included adult–child anthropometrics (weight [kg], body mass index [BMI] including BMI% and *Z*-scores for offspring, waist circumference [cm]) and child diet quality estimated using the 2015 Healthy Eating Index (HEI) from a single random food record. Wilcoxon signed rank was used to compare differences between baseline and 2-week anthropometrics measures and offspring total HEI scores. Qualitative data were analyzed using thematic analysis to understand factors attributed to diet adherence and acceptability.

**Results:**

Eight families enrolled (*n* = 8 adults, *n* = 12 offspring), with 7 families completing the study (12% attrition) and completing 100% of study outcome measures. Adults experienced a median weight loss of − 1.0 kg from baseline to 2 weeks (79.5 kg to 78.5 kg). Offspring had a 24-point increase in total 2015 HEI scores (median difference 50 to 74). Focus group participants (*n* = 4 adults) reported being satisfied with the program and expressed a willingness to continue the diet despite identified barriers.

**Conclusions:**

A whole foods diet that provides dietary support was found to be feasible and acceptable for families at risk for T2D. Future studies are needed to test the effects of the diet on prediabetes outcomes, diet quality, and diet adherence in adults and families.

**Trial registration:**

NCT05483972 at ClinicalTrials.gov. Registered July 25, 2022. https://clinicaltrials.gov/study/NCT05483972?cond=prediabetes&term=whole%20foods%20&rank=1

## Key messages regarding feasibility

- Diet patterns that prioritize whole foods can improve diabetes outcomes and reduce the risk for type 2 diabetes. It is unknown if adults with prediabetes and their offspring are interested in following a whole foods diet pattern to reduce their risk for type 2 diabetes and improve their diet quality.
- This study found that a whole foods diet adapted from the 2020–2025 Dietary Guidelines for Americans framework is both feasible to implement and acceptable for adults with prediabetes and has the potential to improve diet quality in offspring at high risk for diabetes.
- The implications of this study support the acceptance of a whole foods diet in a small and diverse sample of families at risk for type 2 diabetes. A future clinical trial is needed to test the diet’s effects on glycemic control in adults with prediabetes and diet quality improvement in families over a longer duration.

## Background

An estimated 37 million adults have type 2 diabetes (T2D) while an additional 96 million are considered “high risk” for T2D due to having prediabetes [1]. Prediabetes is a condition of impaired fasting glucose and/or impaired glucose tolerance [2] and is a major T2D risk factor. T2D disproportionately affects minority and low-income groups (e.g., non-Hispanic Black, Latino/Hispanic) rendering them medically vulnerable to experiencing multiple co-morbid complications [3]. Moreover, millions of children and adolescents born to a parent affected by T2D are likely to develop the condition at an earlier age [4, 5]. Without treatment, 70% of individuals with prediabetes are projected to develop T2D within their lifetime [6]. To prevent a sequela of early-onset complications and co-morbid conditions linked to T2D (e.g., cardiovascular disease), effective treatment at the onset of prediabetes is required [7].

Dietary approaches have been widely studied for over two decades and are considered a first-line treatment to manage and prevent T2D [8]. Previous T2D diabetes prevention trials primarily used low-fat and calorie-restricted diets to achieve weight loss [9, 10]. However, while these approaches were effective short-term [9, 10], sustained weight loss was often difficult to maintain long-term [11] which resulted in the exploration of other dietary interventions to treat T2D.

Plant-rich eating patterns like the Mediterranean, vegetarian, and certain high-quality carbohydrate-restricted diets that prioritize nutrient-dense whole foods (e.g., fruits, vegetables, unrefined whole grains, beans, legumes, nuts) have been linked to improved glycemic outcomes (e.g., fasting glucose) and a reduced risk for T2D [12–14]. Importantly, the beneficial effect of these diets is thought to be due to their dietary composition and quality (e.g., high fiber, reduced glycemic load) which directly affects glycemia by slowing insulin secretion and reducing postprandial glucose levels and body fat deposition via lipogenesis in the liver and adipose tissue [15]. A U.S.-Style diet pattern (with and without the prioritization of whole foods) and its relationship with prediabetes outcomes has not been studied. Also, while diet patterns like the Mediterranean diet are recommended as part of the treatment approach for prediabetes, studies have failed to capture how individual, family, and cultural dietary preferences influence long-term adoption among U.S. families [16].

One diet pattern that has been understudied is the 2020–2025 Dietary Guidelines for Americans (DGA) framework. The current DGA supports a customizable diet pattern of nutrient-dense foods that also allows for individual/family, cultural, and budgetary considerations to “meet people where they are” [17]. Given the limitations of past diabetes prevention trials which did not prioritize social needs and preferences, a whole foods diet that follows the 2020–2025 DGA framework may be effective for treating prediabetes in adults [9, 10].

To our knowledge, no studies have used a family-centered whole foods diet adapted from the 2020–2025 DGA to treat prediabetes in adults. Therefore, preliminary evidence is needed to determine the feasibility and acceptability of this approach in families at high risk for T2D. We hypothesize that a diet pattern based on the 2020–2025 DGA has the potential to foster family-level support to promote sustained adherence to a whole foods diet without the need for calorie restriction and weight loss and may modify dietary habits in youth to prevent chronic diseases over the life course, including T2D. The objective of this study was to design and deliver a family-centered, non-pharmacological whole foods diet to examine intervention feasibility (i.e., participant recruitment with a 50% family retention rate and ≥ 80% completion of all pre-and post-test study assessments and measures) and acceptability (i.e., qualitative information about diet acceptability from a single focus group session) in adults with prediabetes and their offspring (6–17 years). To assess important secondary patient-centered outcomes, assessment of weight, height, BMI, and waist circumference change over 2 weeks in adults and offspring and change in offspring diet quality during the intervention was evaluated.

## Methods

This prospective single-arm 2-week pre-post experimental study was designed to assess the feasibility and acceptability of a whole foods diet intervention for adults with prediabetes and their biological offspring. The primary goal was to (1) establish the feasibility of recruitment and retention of the study’s target population, (2) assess completion rates of intervention requirements, and (3) assess diet acceptability and factors that may have influenced diet adherence. Examination of secondary patient-centered outcomes included anthropometric assessments in both adults and offspring from baseline to 2 weeks and change in offspring diet quality during the intervention period. The study was approved by the Institutional Review Board at Vanderbilt University, and informed consent was obtained from all study participants. The trial is registered with ClinicalTrials.gov (NCT05483972).

### Participants

A convenience sample of eight families that included an adult parent 25–59 years with prediabetes and up to two biological offspring 6–17 years were recruited from the Nashville TN area between September 2022 to February 2023. Recruitment methods included the use of university email (“Research Notifications Distribution List”), university and medical center recruitment boards, and the online platform ResearchMatch [18].

Eligibility criteria for adults included (1) having a clinician-managed diagnosis of prediabetes based on the American Diabetes Association criteria of either fasting plasma glucose of ≥ 100 mg/dL, hemoglobin A1c (HbA1c) 5.7–6.4%, or 2-h plasma glucose during 75-g oral glucose tolerance test 140 mg/dL to 199 mg/dL that did not require treatment with medications; (2) being at risk for or having overweight or obesity defined as a body mass index (BMI) between ≥ 23 kg/m^2^ to < 40 kg/m^2^; and (3) being a parent to a biological child 6–17 years. Eligibility criteria for offspring included (1) having a body mass index ≥ 5th percentile for age and gender on standardized Centers for Disease Control and Prevention (CDC) growth curves and (2) having an index parent with prediabetes enrolled in the program. Exclusion criteria included (1) past history of T2D (adult), (2) being pregnant or nursing due to increased energy requirements (adult), (3) taking diabetes or weight loss medications (adult and offspring), (4) participating in a weight loss program (adult and offspring), (5) history of bariatric surgery (adult), (6) self-reported engaging in moderate to vigorous daily physical activity (adult), (7) having a special dietary restriction, food allergy, or medical condition that would prohibit eating a variety of foods (adult and offspring), (8) severe mental or neurological impairment (adult), (9) non-English speaking (adult and offspring), and (10) unwilling to follow prescribed diet (adult and offspring).

### Data collection

**Fig. 1:**
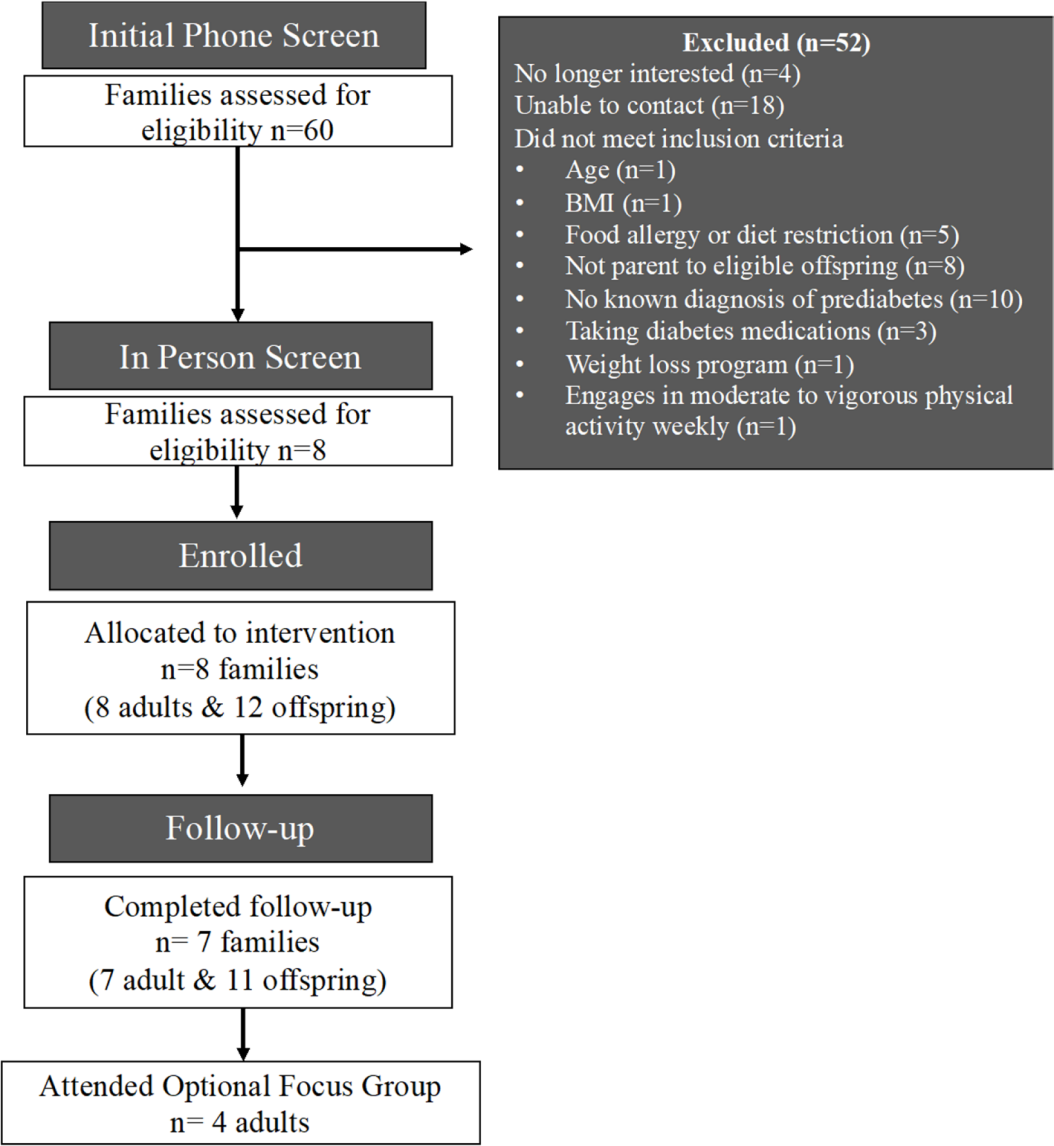
CONSORT diagram of participant flow through the study

A two-part screening phase was conducted and included an initial phone screen to assess preliminary eligibility after which, if eligible, participants were invited for an in-person screening appointment at the Vanderbilt University School of Nursing (VUSN) behavioral clinical laboratory. Participants had their weights and heights measured to confirm their BMI. Adults brought their medical records (e.g., laboratory report, medical diagnosis) for review by a study team member to confirm their prediabetes status. If pre-screening eligibility criteria were verified, participants were enrolled and the start date for the intervention was scheduled. Figure 1 outlines a detailed description of the study screening and enrollment process in line with the CONSORT (Consolidated Standards of Reporting Trials) guidelines.

### Diet intervention

The whole foods dietary intervention consisted of a 2-week controlled feeding period. After enrollment, participants began the intervention on a weekend day following their eligibility confirmation/baseline visit. Delayed enrollment was allowed under special circumstances where religious, cultural, and/or family events coincided with the study start date (e.g., delay during the weeks of Christmas, Chinese New Year). Although the study’s primary objective was to assess outcomes for the enrolled parent and offspring(s), adoption of the diet for the entire household was strongly encouraged due to family involvement being a strong predictor of diet adherence [19–21].

Families received 14 days of menus at the baseline visit. All groceries were delivered using a grocery delivery service (Shipt) and included all ingredients required to prepare each meal and snack to feed up to 4 family members. Three deliveries were scheduled during the 14-day period. If there was not enough food provided to feed the entire family, participants were asked to purchase additional ingredients to supplement the meals. Participants received kitchen tools that were necessary to prepare and cook their meals (e.g., measuring cups, sheet pans, grater, etc.). The study registered dietitian nutritionist coordinated grocery deliveries with participants on an agreed-upon time and date. Menus were developed by our study dietitian using the Nutrition Data System for Research (NDSR) [22], which provides a comprehensive output of diet composition (e.g., macronutrients, calories, food groups). The study dietitian coordinated with the grocery delivery partner in real time to assist shoppers in selecting substitutions for “out of stock” or “unavailable” food items. If participants reported any missing, inedible, or incorrectly delivered food items, the study dietitian placed a new order to replace these items or selected alternative options that closely aligned with the menu’s macronutrient and calorie needs. The study dietitian would coordinate an additional time/date for participants to receive their extra grocery delivery. The study dietitian also offered guidance on how to incorporate substitutions into the menus and assisted participants with how to report these changes on their food checklists. Participants were told that the total time commitment of study was 4.5 h each week (up to 9 h total) plus time to prepare and cook meals (about 1–2 h daily).

Enrolled adults were counseled to strictly adhere to the diet intervention. Menus were designed to be weight-maintaining to limit significant fluctuations in body weight and were based on the adult’s estimated energy requirements [23]. This approach allowed for the preliminary testing of implementing a weight-maintaining diet in adults with prediabetes to assess the feasibility and acceptability of this method before implementation in a larger RCT. Offspring were encouraged to eat ad libitum (i.e., as desired) until they felt full and were asked to follow the diet when at home or to take lunches to school per their family’s preferences. To prevent confusion with the weight-maintenance diet recommendations for adults, the study dietitian counseled parents that they should not restrict their children’s diets. Families were encouraged to eat meals together with limited distractions (e.g., media use, phones, television) since media use is associated with a lower diet quality in children and adults [24, 25].

Adults were counseled by the study dietitian weekly via phone and were responsible for taking the lead on the diet intervention at home or relaying the information to the primary caregiver/spouse/partner in the household responsible for meal preparation/cooking. During meetings with the study dietitian, information about diet instruction, meal preparation/planning, and individual and family goal setting was provided. Offspring were encouraged to participate in meal planning, preparation, and cooking when age appropriate.

The whole foods diet intervention was designed to align with a healthy diet pattern focused on the primary consumption of minimally processed (e.g., fruits, vegetables, meats, eggs, milk, seeds, nuts) and processed (e.g., canned or bottled foods using minimally processed ingredients, cheeses) foods that align with the current Nova Classification System recommendations [26, 27]. All reasonable efforts were made to limit foods classified as “ultra-processed” based on NOVA guidelines in the study menus to include room for more nutrient-dense (e.g., higher quantities of nutrients and minerals) food choices [17, 26, 27]. The whole foods diet incorporated a limited number of “convenience” foods requiring little to no preparation (e.g., bananas, nuts, snack cheeses) to improve diet sustainability and increase the likelihood that participants would be able and willing to continue the diet on their own initiative after study completion. Menus were consciously designed to allow for modifications that included canned/frozen foods to reduce participant costs when possible and aligned with macronutrient nutritional goals outlined by the 2020–2025 DGA for age-sex groups. Macronutrient intake was structured as 25:45:30% energy from protein to carbohydrates to fat. Saturated fat was limited to < 10% of total fat intake, and added sugar totaled < 10% of daily calories. Dairy products included low-fat or fat-free versions [17].

## Outcome measures

### Primary feasibility and acceptability outcomes

For this study, the primary outcomes were the feasibility and acceptability of the whole foods diet intervention. Feasibility was based on the following: (1) successful recruitment of our pre-determined sample size allotment of between 8 and 12 families which was based on budgetary considerations, (2) having a ≥ 50% family retention rate, and (3) ≥ 80% completion of all pre-and post-test study assessments and measures that included questionnaires and dietary recall assessment, anthropometric measures, and weekly meetings with the study registered dietitian during the 2-week study period.

Acceptability of the whole foods diet was based on qualitative findings elicited during the focus group. One 90-min focus group was conducted via Zoom by a trained expert (author C.D.) in collaboration with the Vanderbilt Qualitative Research Core. Open-ended scripted questions were asked using a moderator’s guide that included questions related to (1) experiences participating in the whole foods study, (2) menu and food likes/dislikes, (3) preparing and cooking meals, (4) dealing with food waste, recipe modification, and alternative foods, and (5) continuation of the whole foods diet. Follow-up questions were asked for clarity purposes and to facilitate detailed discussion.

### Secondary patient-centered outcomes

#### Anthropometry measurements

As noted above, weight, height, and waist circumference measurements of adult/child dyads were collected at baseline and study completion at the VUSN behavioral clinical laboratory. Measurements were taken by trained staff. The CDC National Health and Nutrition Examination Survey procedures for accurate BMI and waist circumference measurement were followed [28, 29]. Weight and height were measured with the participant in light clothing without shoes to the nearest 0.1 kg using a calibrated digital scale. Height was measured to the nearest 0.1 cm using a free-standing portable stadiometer (Seca 213). Weight in kilograms divided by the square of height in meters was used to calculate BMI [30], and the CDC BMI-for-age charts for children above 2 years was used to calculate BMI percentiles and *z*-scores [31]. BMI-*z* scores were estimated using an online calculator from the Children’s Hospital of Philadelphia Research Institute using the following measures: weight (kg), height (cm) gender, date of birth, and date measurement was taken (https://zscore.research.chop.edu/calcbmi.php).

#### Dietary intake and adherence

The National Cancer Institute’s ASA24® Dietary Assessment Tool was used to collect a baseline 24-h dietary recall from all participants [32]. Briefly, ASA24 uses a multiple-pass method to collect diet information from the prior 24 h. Trained study personnel administered the recall in-person during the participant’s baseline visit at the VUSN behavioral clinical laboratory to ensure complete dietary intake information was captured prior to participants beginning the intervention. Participants were asked to report all foods and beverages consumed (excluding supplements) from midnight to midnight the previous day. In some instances, a parent or guardian was asked to provide a proxy report of their child’s dietary intake information if the child was unable to complete the recall alone or in its entirety [33]. ASA24 is considered a valid method of reporting dietary intake relative to true intake [34, 35] and has been successfully used in studies that have included older, multiethnic, and low-income adults and children [36–38]. Participants were asked to complete a daily food checklist, similar to a food diary. Food checklist included each days’ meals/snacks which were pre-written on the checklist to reduce participate burden and support accurate reporting of foods consumed. Participants were asked to record all foods and beverages consumed and were encouraged to report any deviations from the menu, including any menu substitutions made by the study dietitian in the event that food substitutes were required. The food checklists allowed participants to report the time and place the meals were eaten and place checkmarks by the foods eaten and their amounts (e.g., ½ chicken salad). If more detailed information was needed or if menu deviations occurred, participants were instructed to report this information on the free-text portion of their checklist and report quantities consumed (e.g., tablespoons, fluid ounces, cups, etc.). Parents were instructed to assist offspring with the checklists and were asked to provide as much detail as possible. Food checklists were not statistically analyzed to estimate diet adherence; however, they were collected after the 2-week follow-up visit and reviewed by the PI and study dietitian to identify menu items that exhibited poor adherence.

#### Diet quality

The 2015 Healthy Eating Index (HEI-2015), a scoring system (0–100 points) for ages ≥ 2 years that can estimate how well an individual’s diet aligns with major DGA recommendations [39], was used to estimate baseline diet quality scores of adults and offspring and post-intervention scores in offspring only. HEI-2015 was used because the newest HEI-2020 was not released until late July 2023 and was not available at the time the data were analyzed. A score of 100 indicates perfect alignment with the dietary guidelines, an 80–51 score indicates “needs improvement,” and < 51 score indicates a “poor diet” [40, 41]. To calculate HEI-2015 scores, diet information was collected from the baseline 24-h diet recall imported into ASA24 (adults and offspring) and from a random and completed (i.e., full-day) food checklist entry reported within the 14-day study period (offspring). Data collected from the food checklists were entered manually into the ASA24 software by a trained research assistant familiar with the program.

#### Estimated energy requirements (EER)

EERs were calculated for adult participants at baseline to determine caloric requirements for weight maintenance during the intervention. An online EER calculator (omnicalculator.com) that included EER formulas developed by the Institute of Medicine for males and females was used that accounted for age, sex, weight, height, and physical activity. An activity factor of 1.11 for males and 1.12 for females was applied to all estimates. This is a validated method for estimating an individual’s caloric requirements to achieve energy balance and has been tested across a wide range of genders and racial/ethnic groups [23]. The EER calculations were used by the study team and dietitian to provide adults with the appropriate menus that aligned with their specific calorie requirements to maintain their baseline weight. This method allowed the study dietitian to provide simple modifications to match each adult participant’s specific dietary needs (e.g., 2200 kcal diet provided to an adult whose EER was 2285 kcal per day).

#### Other questionnaires

Additional data collected included sociodemographic characteristics, child food patterns and eating behaviors (youth risk behavior surveillance questionnaire) [42], parent diet habits via an 8-item screener [43], and food insecurity via the 6-item household food insecurity screener [44].

## Data analysis

### Quantitative analyses

Given the preliminary nature of this study, a sample size justification was not applicable. Feasibility targets were defined a priori and included ≥ 50% family retention rate and ≥ 80% completion of all pre-and post-test study assessments and measures (i.e., questionnaires, anthropometric measures, weekly attendance with registered dietitian). Acceptability of the whole foods diet was based on qualitative findings elicited during the focus group.

Data were analyzed using the SPSS software (version 29.0.0; IBM Corp) with the exception of HEI-2015 data which was analyzed using SAS On Demand (version 3.81, SAS Institute Inc.) using codes and macros provided on the ASA24 researcher website (https://epi.grants.cancer.gov/hei/sas-code.html). Total baseline HEI-2015 scores for adults and offspring, and HEI-2015 scores for offspring during the intervention period were analyzed using the simple HEI scoring algorithm-per-day macro. All data were transferred to SPSS as part of the final analysis.

Descriptive statistics were generated and included medians, interquartile ranges [IQR], and frequency counts to describe the study population, baseline dietary macronutrient intake, anthropometric measurements, and diet quality scores (via HEI-2015). Diet acceptability was based on participant feedback provided during the 90-min focus group session conducted at study completion.

Due to having a small sample size and non-normally distributed data, Wilcoxon signed rank was used to compare differences between pre-intervention and post-intervention outcomes for anthropometrics measurements of adults and offspring (within-group comparisons) and HEI-2015 total scores (offspring-only). Per CONSORT 2010 guidelines, median and 95% confidence intervals (95% CI) estimates were provided in place of formal hypothesis testing [45].

### Focus group analyses

The focus group was conducted, transcribed, coded, and analyzed by the Vanderbilt Qualitative Core following the COREQ [46] guidelines, an evidence-based qualitative methodology. Questions were generated to elucidate potential barriers and/or facilitators associated with participation in the whole foods intervention. Themes were generated to discuss cost, healthy food availability, time requirements to eat healthy, feasibility of participating and including children in the process of planning/cooking, and level of diet support needed to maintain diet adherence.

To establish coding reliability, experienced qualitative coders independently coded the first 100 lines of the focus group transcript. Coding was then compared, and all discrepancies were resolved. After establishing reliability in use of the coding system, the coders divided and independently coded the remaining lines. Each line was treated as a separate quote and was assigned up to 13 different codes. Coded transcripts were combined and sorted by code. Quotations and codes were managed using Microsoft Excel 2016 and SPSS version 28.0.

The analysis consisted of interpreting the sorted, coded quotes and identifying higher-order themes using an iterative inductive-deductive approach [47]. The goal of the iterative inductive-deductive approach was to develop a conceptual framework that is theoretically informed while integrating resultant content from the qualitative data. Deductively, the analysis was guided by the Social Cognitive Theory [48] wherein behavior is influenced by the interaction of personal factors, environmental factors, and behavioral factors. Inductively, the framework’s content was derived from the qualitative data.

## Results

### Feasibility and acceptability

Eight adults and 12 offspring completed baseline testing. One family (1 adult/1 child) asked to stop the diet because they reported they were unable to adhere to the intervention as intended. In total, 7 families (7 adults, 11 offspring) completed baseline and 2-week follow-up testing. Participants completed 100% of baseline data collection requirements (i.e., anthropometric assessment, dietary recall collection, survey completion). Among the 18 participants who completed the study, 100% of adult parents participated in all scheduled calls with the study dietitian and 100% of adults and offspring completed 2-week follow-up anthropometric testing within 14 days of study completion. Information about dietary adherence was not statistically analyzed but was reviewed by the PI and study dietitian to assess overall adherence to the diet protocol.

Participants who completed the focus group session (4 total) reported being satisfied with the diet and expressed a willingness to continue the diet on their own despite identified barriers experienced during the 2-week intervention period.

### Baseline characteristics

Baseline characteristics are described in Table 1. Among adults, the average age was 47 years, and the majority of participants were female (87.5%), non-Hispanic White (37.5%), and considered to have obesity (31.3 kg/m^2^). At baseline, the estimated mean caloric intake was 1982 kcals/day, and the average total HEI diet quality score was 59. Among offspring, the average age was 10 years, and the majority of participants were female (58.3%), non-Hispanic White (41.7%), and considered to be below the 85% of BMI-for-age. Their baseline estimated mean caloric intake was 1620 kcal/day, and their average total HEI score was 50.

**Table 1:** Overall baseline characteristics of 8 enrolled families: adults 25–59 years with prediabetes and offspring 6–17 years

Adult characteristics (*n* = 8)	*n (%)*	Offspring characteristics (*n* = 12)	*n (%)*
**Age, median (IQR)**	47 (41–54)	**Age, median (IQR)**	10 (9–15)
**Sex**		**Sex**	
Female	7 (87.5%)	Female	7 (58.3%)
Male	1 (12.5%)	Male	5 (41.7%)
**Race/ethnicity**		**Race/ethnicity**	
Asian	2 (25.0%)	Asian	2 (16.7%)
Non-Hispanic Black	2 (25.0%)	Bi-racial^a^	2 (16.7%)
Non-Hispanic White	3 (37.5%)	Non-Hispanic Black	2 (16.7%)
Other race^a^	1 (12.5%)	Non-Hispanic White	5 (41.7%)
**Anthropometrics**		Other race	1 (8.3%)
Weight kg, median (IQR)	86.3 (72.3–95.3)	**Anthropometrics**	
BMI kg/m^2^, median (IQR)	30.4 (25.2–37.5)	Weight kg, median (IQR)	48.3 (36.3–64.3)
BMI weight status		BMI kg/m^2^, median (IQR)	19.4 (17.4–24.8)
Normal (23–24.99 kg/m^2^)^b^	2 (25.0%)	BMI percentile, median (IQR)	78 (53.5–96.7)
Overweight (25–29.99 kg/m^2^)	2 (25.0%)	BMI *z*-score, median (IQR)	.46 (− .24–1.80)
Obese (30–39.99 kg/m^2^)	4 (50.0%)	BMI weight status	
Waist circumference cm, median (IQR)	101.1 (88.6–110.1)	Normal (5th to < 85th %)	8 (66.7)
**Caloric intake, median (IQR) kcals**	1982 (1735–2720)	Obese (≥ 95th %)	4 (33.3)
**HEI-2015 total score, median (IQR)**	59 (47–66)	Waist circumference cm, median (IQR)	74.1 (65.6–79.3)
**Nutrition assistance**^**c**^		**Caloric intake, median (IQR) kcals**	1620 (1381–2281)
No	8 (100.0%)	**HEI-2015 total score, median (IQR)**	50 (41–64)
**Marital status**			
Married	5 (62.5%)		
Divorced, separated, widowed	3 (37.5%)		
**Education**			
College or associate degree	4 (50.0%)		
Postgraduate/professional degree	4 (50.0%)		
**Household income**			
$35,000 to $49,000	1 (12.5%)		
$50,000 to $74,999	3 (37.5%)		
$75,000 to $99,000	1 (12.5%)		
> $100,000	3 (37.5%)		
**Insurance type**			
Private insurance	6 (75.0%)		
Medicaid	1 (12.5%)		
Government sponsored^d^	1 (12.5%)		
**Employment status**			
Full-time	7 (87.5%)		
Retired	1 (12.5%)		

Values are frequencies (%), unless otherwise noted

BMI was calculated as weight in kilograms dived by the square of height in meters. SD, standard deviation; %, percentile

*Abbreviations*: *BMI* Body mass index, *HEI* Healthy Eating Index, *IQR* Interquartile range, *kcals* Kilocalories

^a^Other includes Middle Eastern and bi-racial includes Asian/Non-Hispanic White

^b^BMI category of 23 kg/m^2^ in Asian adults is considered “overweight”

^c^Indicates Supplemental Nutrition Assistance Program participation only

^d^Includes coverage under the Affordable Care Act

### Secondary patient-centered outcomes

Changes in anthropometric measures from baseline to follow-up at 2 weeks for adults and offspring are shown in Table 2. In adults, an overall reduction in weight (kg) was noted (79.5–78.5 kg; median difference − 1.0 kg; 95% CI: − 2.4, 0.2). There were minimal changes in adult BMI or waist circumference between the baseline and 2-week follow-up visit.

**Table 2:** Baseline and 2-week anthropometry change for adults (25–59 years) and offspring (6–17 years)

	**Adults (*n* = 7)**	**Median [IQR]**	**Median [95% CI]**	**Offspring (*n* = 11)**	**Median [IQR]**	**Median [95% CI]**
Weight (kg)	Baseline Week 2 Median difference	79.5 [93.9–71.1] 78.5 [94.2–68.8] − 1.0	− 1.2 [− 2.4, 0.2]	Baseline Week 2 Median difference	43.5 [63.4–35.9] 43.7 [62.6–34.7] 0.2	− 0.3 [− 1.2, 0.3]
BMI (kg/m^2^)	Baseline Week 2 Median difference	28.3 [24.5–36.4] 28.1 [23.0–36.9] − 0.2	− 0.2 [− 0.9, 0.2]	Baseline Week 2 Median difference	18.7 [17.3–24.8] 18.3 [17.3–24.6] − 0.4	− 0.1 [− 0.4, 0.1]
Waist circumference (cm)	Baseline Week 2 Median difference	95.5 [87.0–108.0] 95.9 [83.7–109.8] 0.4	− 2.5 [− 5.3, 0.4]	Baseline Week 2 Median difference	73.3 [65.4–76.1] 68.5 [58.2–77.8] − 5.6	− 1.7 [− 4.7, 1.5]
BMI percentile (%)				Baseline Week 2 Median difference	74.0 [52.0–96.7] 62.8 [32.1–95.4] − 11.2	− 3.1 [− 29.9, 0.4]
BMI *z* score				Baseline Week 2 Median difference	0.3 [− 0.3–1.7] 0.3 [− 0.5–1.7] 0.0	− 0.1 [− 0.2, 0.0]

*Abbreviations*: *BMI* Body mass index, *CI* Confidence interval, *cm* Centimeters, *kg* Kilograms

In offspring, minimal differences were observed for all anthropometric measures (Table 2). However, increases in diet quality scores were noted, and offspring had a median 24-point increase (95% CI: 4.5, 33.0) in their HEI-2015 scores from baseline (Table 3).

**Table 3:** Baseline and week 1 or 2 diet quality score change in offspring (6–17 years)

	**Offspring (*n* = 8)**	**Median [IQR]**	**Median [95% CI]**
HEI-2015	Baseline Week 1 or 2 Median difference	50 [40–64] 74 [65–81] 24	18.5 [4.5, 33.0]

*Abbreviations*: *CI*, Confidence interval, *HEI-2015* Healthy Eating Index 2015

### Details of qualitative focus group discussion

**Fig. 2:**
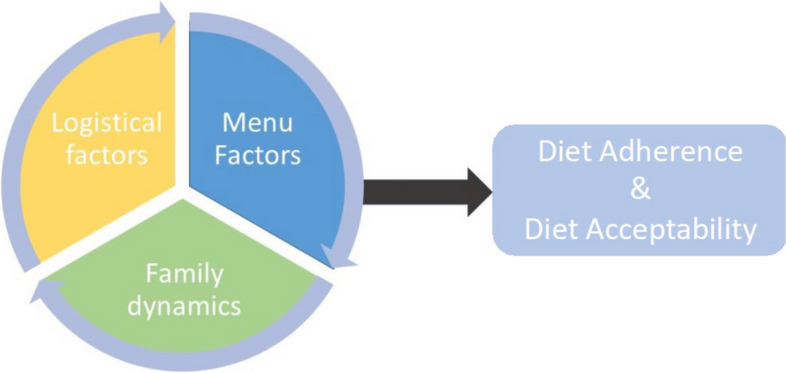
Conceptual framework of influential factors affecting diet adherence and acceptability

Five participants who completed the intervention signed up for the optional 90-min focus group session at the end of the study. Four female adults (median age 52 years) that included 2 non-Hispanic White, 1 non-Hispanic Black, and 1 non-Hispanic Asian participants participated in the 90-min session. Three themes emerged from the focus group: (1) menu factors, (2) family dynamics, and (3) logistical factors. Figure 2 illustrates the interaction between person-level and contextual factors that influence adherence to and acceptability of the whole foods diet. Representative quotes for each theme and specific to the overall adherence and acceptability of the diet are reported in Table 4.

**Table 4:** Focus group responses related to qualitative themes

Theme	Representative quotes
Menu factors	“…and I think some spice, maybe we can skip that”
	“And what I found was that my family ate the things that were most familiar”
	“We are frozen food, put it in the oven, go do your homework, get in the shower. It was so vastly different and I haven’t said this yet, but my kids really enjoyed the process of cooking and learning to cut an onion and learning to cut bell pepper”
Family dynamics	“I had a seven-year-old and a 10-year old participating and there was my seven-year old didn’t even try. He was just not going to, there wasn’t anything on any of the days that he was willing to give a shot to”
	“And then I had actually had a day off where I just meal prepped for basically the rest of the week”
	“…I get off at 5:30, 6 o’clock some evenings. So by the time I had prepared the food it was pretty late quite often and I had to become pretty creative with preparing the foods”
	“My daughter [age 10], actually, the salmon on farro, basically made the entire meal”
	“my refrigerator was not prepared. We too had to clean out everything, take out everything that we could from the freezer to make room for things and still had a lot on our countertops”
	“I can’t then spend an hour and a half cooking” “So it is very easy. And now it is not like a jambalaya. I take 30 min to prepare”
Logistical factors	“So if I don’t finish it before it goes bad and what am I going to add it to a spoon full of her lunch, maybe it that to me and I feel bad throwing out things because we’re just never going to get to it”
	“It’s definitely more cost-effective to buy the ingredients and make the meals. It is literally just the ease factor”
Overall diet acceptability and adherence	“with this meal plan I start liking salad […] So I think it definitely changed my life”
	“And so I don’t buy other bread now. I just buy the 12 grain awesome bread”
	“…I switched to Dr. Pepper zero and so I don’t drink, Dr. Pepper doesn’t taste as good anymore.” and “it has changed the amount of sugar I consume because products that are pre-made are now too sweet”

#### Menu factors

Participants were asked to discuss how they felt about specific menu items, modifications needed, and how the menu compared to their standard pre-intervention diet. Participants reported enjoying the menu overall but did identify areas for improvement. For example, one participant recommended using fewer spices and limiting strong flavors to improve adherence among their children. Another participant reported that it was easier to cook with familiar food items, while others reported it was helpful to batch cook meals or reuse ingredients to streamline the process.

Participants compared the menu selections with the typical meals their family ate. There were varying responses, with some noting it was similar to their normal diet and others reporting that it was quite different. Most participants reported favoring the menu in comparison to their standard diet and expressed a desire to continue eating similarly.

#### Family dynamics

Participants discussed personal characteristics that impacted their experiences engaging with the whole foods intervention such as the age of their children, their schedules and obligations, their knowledge of cooking, the physical features of their households, and the amount of time they took to prepare meals. Most participants indicated that the ages of their children influenced what foods their children were willing to eat. Some participants explained that their younger children were “picky eaters” and refused to eat certain foods on the menus, especially spicy foods. Other participants mentioned how personal schedules and obligations impacted their ability to execute the diet. Many reported struggling to find enough time to cook meals and fulfill their schedule obligations. One participant tried to mitigate this issue by cooking on her day off. However, another participant reported she needed to stay up late to prepare meals.

Participants discussed their family members’ contributions towards food preparation throughout the duration of the program. Most participants indicated that their families were involved with food preparation which facilitated positive diet experiences. Participants also shared how knowledgeable they felt about cooking and explained how this personal knowledge affected their ability to prepare meals throughout the program. Some participants in the group indicated that preparing meals in the program may be difficult for those with limited knowledge. They also discussed how well prepared their kitchens were to prepare, cook, and store the meals The complexity of recipes and the subsequent amount of time they took to prepare meals was also discussed. Some participants said that certain meals were too complex and/or took too long to prepare. These participants indicated that they felt overwhelmed with the amount of time to cook meals and having enough time to fulfill other obligations. However, participants also reported that some meals were easier to follow and took less time to make.

Responses conveyed that participants’ unique personal characteristics and experiences impacted their ability to implement the diet intervention. Personal characteristics such as increased family involvement, older children, and established cooking knowledge facilitated positive experiences completing the diet. However, younger children with strong food preferences, limited cooking knowledge, demanding schedule obligations, and increased complexity and meal preparation time produced barriers for participants during the program.

#### Logistical factors

Participants identified several logistical factors that influenced their adherence to the whole foods program and their ability to continue implementing it. Commonly, these factors included the management of excess food and the cost of groceries. One participant reported being so concerned about the food waste that she reached out to the study dietitian who was able to adjust the amount of excess food.

Participants discussed how external factors such as cost would impact their ability to continue with the diet. Participants expressed that the cost of food would not be a barrier that prevents them from continuing the diet. One participant explained how produce is relatively affordable in comparison to processed foods.

#### Overall diet acceptability and adherence

Menu factors, family dynamics, and logistics all contributed to each family’s adherence to the intervention as described in the quotes above. Additionally, these factors influenced the participants’ willingness to continue aspects of the intervention after the study protocol was complete. Some participants described how the meal plan expanded the types of foods they eat. Other participants reported making healthier food swaps after the intervention was over or noting a change in their taste preferences.

## Discussion

This study found that a whole foods diet adapted from the 2020–2025 DGA is both feasible and acceptable for adults with prediabetes and their offspring. The study had a high family attrition rate (12%) with 100% of participants completing all required surveys, anthropometry, and dietary recall collections at the baseline and 2-week follow-up visit. Focus group participants (*n* = 4 adults) reported being satisfied with the program and expressed a willingness to continue the diet protocol despite identified barriers. Additionally, offspring experienced an increase in their diet quality scores while participating in the intervention.

This is the first known study to test the feasibility and acceptability of a whole foods diet intervention adapted from the 2020–2025 DGA for adults with prediabetes and their offspring. The DGA framework was used in this study due to its major focus on individual/family needs and preferences to support customization to “meet people where they are” [17]. Interestingly, numerous comments emerged during the study’s focus group illustrating the importance of culture, family, and social factors (e.g., food costs) for diet acceptance and adherence. For example, cultural and family factors contributed to the like and/or dislike of certain menu items. Participants reported that if a menu item had strong flavors from spices, their offspring were often unwilling to eat the meal which resulted in some participants removing spices from the menus entirely. Alternatively, one participant commented that her family members were more likely to eat familiar menu items. There were also comments about costs associated with continued adherence to the whole foods diet. For example, participants in the focus group unanimously reported that time to prep the food was a major barrier rather than food cost. However, participants in this study were all employed and did not report the use of nutrition assistance. Therefore, these qualitative findings may not be generalizable to low-income families or those who experience food insecurity. Nonetheless, the focus group comments provide a rich description of how family, cultural, and social factors directly influenced the acceptability and adherence to the whole foods diet at the individual and family level. Future studies should consider including these factors in the design and delivery of diet interventions for families at high risk for T2D.

One unique feature of this study was the use of a non-calorie-restricted diet to support weight maintenance. Though weight loss was not encouraged, adults did experience a median weight reduction of − 1.0 kg at the 2-week follow-up which translates into a 1.3% total loss. Weight loss ≥ 7% has been shown to improve glucose tolerance and reduce the risk for T2D [9]. Yet, the percentage of weight lost among adult participants in this study was well below the ≥ 7% threshold. These findings are comparable to similar studies that used non-calorie-restricted weight maintenance diets, including those with longer intervention periods of 8 weeks or greater [49, 50].

Given that this was a feasibility study, the intervention was not powered to test the direct (i.e., weight-independent) effects of a whole foods diet on diabetes outcomes in adults. Yet, a growing body of evidence suggests that a high-quality healthy diet pattern can directly influence diabetes outcomes without the need for significant weight loss. For example, in an 8-week cross-over study of adults (< 65 years) with early-stage T2D, adherence to a weight-maintenance Mediterranean-style diet that incorporated smaller and more frequent meals (6 per day) was associated with improved glycemic control [50]. In another cross-over study of adults (≥ 18 years) with prediabetes and T2D, adherence to either an ad libitum Ketogenic or Mediterranean-style diet reduced hemoglobin HbA1c by 12 weeks. The study reported that the dietary effects were attributed to the use of high-quality foods that limited refined grains and added sugars [12]. Lastly, evidence from the prospective Prevención con Dieta Mediterránea (PREDIMED) RCT of older adults with cardiovascular risk found that adherence to a non-calorie-restricted Mediterranean diet resulted in a 53% reduction in T2D incidence over 4 years, even after controlling for weight loss and physical activity [51, 52]. Though the underlying mechanisms responsible for these metabolic effects are not fully understood, diet patterns that prioritize whole foods often have a lower glycemic load (e.g., brown rice, unprocessed meats, berries, etc.) which results in lower postprandial glucose and insulin secretion [53]. In contrast, diets that allow for greater intakes of carbohydrate-rich foods that do not limit refined or processed products (e.g., desserts, sugar-sweetened beverages, potato products) have been linked to a cascade of metabolic disorders leading to excess adipose tissue deposition in lean muscle tissues, increased postprandial hyperinsulinemia, and a reduction in metabolic rate that coincides with increased hunger [54]. Based on this body of research, a future study is planned to investigate how the quality and composition of a whole foods diet may directly influence glycemia in adults with prediabetes in the absence of significant weight loss (< 7%).

This study also compared changes in diet quality of offspring during the 2-week intervention. A total of 8 offspring experienced a median 24-point increase in HEI scores compared to baseline. The whole foods diet had an almost perfect 2015 HEI score of 98 (day 1 2000 cal menu analyzed indicating subgroups scores for total dairy, sodium, and saturated fat at 9.0, 9.5, and 9.4 respectively) which likely drove the increase in offspring HEI who complied with the diet [40]. Another factor attributed to the high HEI scores may, in part, be due to the family-centric nature of the intervention that encourages diet uptake at both the individual and family unit. Studies have consistently found that parental modeling of healthy diet behaviors and frequent meals consumed at home are major contributors to high diet quality scores in children and adolescents. Family-based interventions also promote sustained dietary behavior changes in both adults and offspring, with benefits for children and adolescents often sustained into adulthood [55, 56]. The qualitative findings from this study support the importance of family engagement in promoting healthy dietary behaviors among youth. It is possible that a family-centered whole foods diet that aligns with current DGA recommendations may offer multigenerational protection against T2D through improved diet quality in at-risk families, though more studies are needed to establish this possible association.

This study has some limitations. First, the study was not adequately powered and only study completers were analyzed. Therefore, we were unable to detect any significant effects of a whole foods diet on anthropometric change among study participants. Given the small sample size, a future intervention is planned to assess the feasibility and acceptability of the intervention in a larger sample (~ 30 families) and over a longer duration (i.e., 12 weeks) that will allow for the assessment of the intervention’s preliminary effectiveness on body adiposity outcomes (e.g., BMI, weight, waist circumference) and potential for sustainability at 3 months. Second, participants did not strictly adhere to the diet as recommended which was noted during review of food checklists. Though not statistically analyzed, review of each participant’s food checklist was evaluated to understand which menu items exhibited low adherence. This information will be used to modify the current menus in a future study to support better adherence. Also, during the focus group session, participants reported that providing food helped facilitate diet adherence among the family. However, participants also discussed common barriers that reduced diet adherence including (1) feeling too full to eat the entire meal at a single sitting, (2) not liking certain menu items, and (3) not having enough time to follow the menus. Offspring adherence was lower when compared to adults, and common barriers reported by parents that influenced diet adherence included (1) “dislike” of the menu items, (2) having a “picky eater”, or (3) unwillingness to try new foods. In a future study, menus will be adapted based on participant comments and child-friendly options will be incorporated into the diet to promote offspring uptake and adherence. We will also incorporate both qualitative and quantitative assessments in our future study to understand more about factors that influence acceptability of a whole foods diet for families. Lastly, diet quality was estimated in adults and offspring during our single, in-person baseline visit using one 24-h diet recall. During the intervention period, a single random and completed food checklist self-recorded during the 2-week study period was used. Standard practice is to estimate diet-quality using 3 non-consecutive diet recalls; however, given budgetary limitations and our short study time frame, we were unable to collect multiple dietary recalls at baseline. As such, this may have resulted in an overestimation of HEI scores. Also, about half of the offspring food checklists had partially missing or incomplete data limiting the dietary information available. This made it difficult to collect 3 days of complete dietary information to estimate an average HEI score across multiple days.

## Conclusion

The findings from this study are encouraging and support the need for continued investigation of a whole foods diet for the treatment of prediabetes in adults. Future studies are needed to test the effects of the diet on prediabetes outcomes in adults and diet quality in families. Future studies are also needed to assess possible barriers and/or facilitators of long-term diet adherence in real-world settings where food provisions and dietetic consultation are not applicable or feasible.

## Data Availability

The datasets used and/or analyzed for the study are available from the corresponding author on reasonable request.

## Abbreviations

ASA24: Automated Self-Administered 24-h
BMI: Body mass index
CDC: Centers for Disease Control and Prevention
CONSORT: Consolidated Standards of Reporting Trials
DGA: Dietary Guidelines for Americans
EER: Estimated energy requirements
HbA1c: Hemoglobin A1c
HEI: Health Eating Index
NDSR: Nutrition Data System for Research
PREDIMED: Prevención con Dieta Mediterránea
RCT: Randomized controlled trial
T2D: Type 2 diabetes
VUSN: Vanderbilt University School of Nursing
